# Innovative therapeutic approach in becker muscular dystrophy. From murine model to focal muscle vibration: a case report assessment through gait analysis

**DOI:** 10.3389/fresc.2026.1801610

**Published:** 2026-05-14

**Authors:** Filippo Camerota, Claudia Celletti, Chiara Bagnardi, Alessia Rampon, Federico Zangrando, Marco Paoloni, Guido Maria Filippi, Manuela Minozzi, Massimiliano Mangone, Enrico Bertini

**Affiliations:** 1 Physical Medicine and Rehabilitation Division, Umberto I University Hospital of Rome, Rome, Italy; 2 Department of Life Sciences, Health, and Health Professions, Link Campus University, Rome, Italy; 3 Department of Anatomical and Histological Sciences, Legal Medicine and Orthopedics, Sapienza University, Rome, Italy; 4 Department of Neuroscience, Università Cattolica del Sacro Cuore, Rome, Italy; 5 Department of Human Science, Link Campus University, Rome, Italy; 6 Unit of Muscular and Neurodegenerative Diseases, Bambino Gesù Children’s Hospital, IRCCS, Rome, Italy

**Keywords:** focal muscle vibration, muscle dystrophy, neurocognitive exercise, proprioception, rehabilitation

## Abstract

**Background/objectives:**

In patients with Becker Muscle Dystrophy, muscle spindles show slight morphological alterations and in mouse models' neuromuscular spindles are preserved but show functional alterations, such as increased discharge at rest and greater response to low-frequency sinusoidal vibrations. In muscular dystrophies, the response to vibratory stimulation suggest that muscle spindles remain functionally active despite extrafusal degeneration, supporting the use of vibration as a co-rehabilitative intervention. The aim of this clinical case was to qualitatively and quantitatively analyze the functional response of a patient with Becker Muscle Dystrophy treated with focal muscle vibration.

**Methods:**

A 43-years-old man with diagnosis of Becker Muscular Dystrophy was treated with two cycles of focal muscle vibration to the lower limbs and between them he underwent 4 sessions of neurocognitive exercises. Patient was evaluated through gait analysis and specific clinical scales.

**Results:**

Following the treatment period, the patient showed an overall improvement in the scores of the assessment scales. The gait analysis assessment showed a general improvement in the parameters following both treatment cycles, but the data showed that the second treatment, which was longer than the first one, gave more satisfactory and long-lasting results.

**Conclusions:**

Proprioception emerges as an essential component of motor control. In muscular dystrophies, the integration of current pharmacological and genetic strategies with rehabilitation strategies aimed at preserving and enhancing proprioceptive function represents a promising prospect for improving the quality of life of patients with neuromuscular diseases.

## 1 Introduction

Becker muscular dystrophy (BMD) is a recessive X-linked hereditary neuromuscular disorder caused by partial mutations in the dystrophin gene. Unlike Duchenne muscular dystrophy (DMD), which is characterized by a complete absence of the protein, BMD is marked by the presence of dystrophin, but in reduced or partially functional form. This results in a less severe but still progressive clinical course, with onset generally in adolescence or young adulthood and progressive deterioration of muscle function, leading to variable motor deficits and disabilities. The variants responsible for BMD include in-frame deletions of one or more exons (the most common), in-frame duplications and, less frequently, missense variants, small ins/dels, intronic variants and nonsense variants (1, 2). In longitudinal BMD cohorts, deletions ending at exon 51 and the deletion of exon 48 are associated with better functional performance, while groups with del 45–48 and especially del 45–47 show lower average performance and greater variability on motor scales (NSAA, 6MWT, timed tests) (2). The management of these patients therefore requires a multidisciplinary approach, aimed at integrating pharmacological, genetic and rehabilitative treatments (3).

In recent years, scientific research has focused particular attention on the role of neuromuscular spindles, structures that are fundamental for proprioceptive regulation and fine motor control. Studies conducted on mouse models of DMD (mdx) have shown that, although the morphology and number of muscle spindles are largely preserved, significant functional alterations are observed, including an increase in resting discharge and greater sensitivity to low-frequency sinusoidal vibrations (4). These data have fueled the hypothesis that proprioception, although partially compromised by the dystrophic context, may still represent a viable therapeutic target. The translational perspective highlights how the increased resting discharge of the afferent spindle pathway can increase tone/stiffness and potentially accelerate the degeneration of extrafusal fibers; hence the idea of implementing proprioceptive rehabilitation interventions or targeted modulations of mechanosensitive channels (e.g., PIEZO2) as possible adjuvant strategies (5). PIEZO2 is selectively expressed in the proprioceptive neurons of the dorsal root ganglion and is the main ion channel responsible for converting mechanical stimuli into electrical signals at the spindle sensory terminals (6). Genetic and functional evidence shows that the inactivation of PIEZO2 abolishes proprioceptive mechano-transduction, markedly compromising postural control and motor coordination (7). From a clinical point of view, loss-of-function variants in PIEZO2 result in human phenotypes characterized by proprioceptive deficits, sensory ataxic gait, scoliosis and arthrogryposis, confirming the non-redundant role of the channel in the somatosensory system (6). Furthermore, in mouse models, the removal of Piezo2 from proprioceptive neurons not only alters sensory function but is also associated with defects in skeletal development and integrity, highlighting a functional neurosensory-bone axis (8).

Focal Muscle Vibration (FMV) is a non-invasive rehabilitation technique that works by selectively stimulating neuromuscular spindles (9). Through the administration of localized vibratory stimuli, it is able to modulate proprioceptive activity and facilitate muscle activation. In various fields, such as central and peripheral neurological disorders, FVM has shown potential in improving postural control, coordination and movement efficiency (10). In the context of muscular dystrophies, scientific literature suggests that, despite extrafusal degeneration, proprioceptive function remains partially intact, thus opening up the possibility of targeted rehabilitation interventions (11).

Interest of FVM in muscular dystrophies therefore stems from the hypothesis that selectively stimulating the proprioceptive component can support and enhance residual motor function, effectively integrating with other therapeutic strategies currently available. This approach is part of a broader rehabilitation medicine approach, which aims not only to slow functional decline but also to improve patients' quality of life by maintaining their independence.

This study aims to analyze, through a clinical case, the impact of focal muscle vibration in a patient with BMD. The analysis is conducted through standardized clinical assessments, such as strength and functional scales, and through gait analysis, a tool of particular relevance for objectively quantifying the effectiveness of treatment. The results obtained allow us to discuss the potential role of FVM as a rehabilitation co-intervention, capable of enhancing residual functions and offering new therapeutic perspectives in a field that is still largely unexplored.

Ultimately, the introduction of focal muscle vibration in the treatment of DMB represents an innovative opportunity to enrich the rehabilitation arsenal available to clinicians, paving the way for future studies aimed at confirming its effectiveness and defining standardized application protocols.

## 2 Materials and methods

A 43-years-old male patient was evaluated and treated. His history started with an incidental finding of elevated CPK and LDH levels when he was a child, in the absence of symptoms, with no apparent clinical significance. The patient was a warehouse worker in a packaging materials manufacturing company for about 13 years; in 2017 he reported the onset of symptoms: initially muscle weakness in his lower limbs, followed by reduced exercise tolerance with a tendency to fatigue and reduced endurance. Over time, progressive postural instability, loss of balance and repeated falls were perceived. At the time of our first contact, the patient reported limited walking autonomy for medium distances, with marked difficulty in climbing stairs and walking on uneven ground, to the point of requiring visual assistance to limit the incidence of falls. In 2022, he underwent EMG/ENG of the AAII, with normal results, in the absence of signs attributable to conduction defects/deficits. In the same year, initial imaging investigations were also performed using MRI of the lumbosacral spine, which documented, in association with osteoarthritic phenomena with multiple discopathy, widespread adipose degeneration of the paraspinal muscles. The definitive diagnosis came in October 2024, following a new genetic test for myopathies and MLPA (Multiplex Ligation-dependent Probe Amplification) for dystrophin: “Deletion of exons 45–48 of the DMD gene in a hemizygous condition, resulting in the production of a partially functional protein. The result obtained is indicative of the clinical diagnosis of Becker Muscular Dystrophy.” The patient first came to our attention at the Rare Diseases Clinic of the Policlinico Umberto I in February 2025, with the aim of identifying and initiating appropriate rehabilitation treatment.

### 2.1 Methods

The patient was clinically and instrumentally evaluated at several time points: upon arrival at the clinic (T0), at the end of the first cycle of focal vibrations (T1), two months after T1 following four physiotherapy sessions (T2), an additional two months after T2 immediately before the start of the second vibration cycle (T3), at the end of the second vibration cycle (T4), and finally during a check-up two months after T4 (final follow-up) (T5).

#### 2.1.1 Assessment tools

The following scales have been used for a multidimensional monitoring:

- Fatigue Assessment Scale (FAS): The Fatigue Assessment Scale (FAS) is a self-assessment tool developed to quickly and easily measure the subjective perception of fatigue in patients with chronic, neurological and neuromuscular diseases. It consists of 10 items with responses on a five-point Likert scale (from 1 = “never” to 5 = “always”), which investigate both physical and mental fatigue. The total score ranges from 10 to 50, with higher values indicating greater symptom severity.
- Short Form Health Survey (SF-36) is a multidimensional self-assessment tool for health-related quality of life, widely used in clinical and research settings. The questionnaire comprises 36 items divided into eight domains: physical functioning, role limitations due to physical health, bodily pain, perception of general health, vitality/energy, social functioning, emotional well-being, and role limitations due to emotional problems. The scores for each domain range from 0 to 100, with higher values indicating better health. Its validity and reliability make it a standardized reference tool in chronic diseases.
- The Medical Research Council Scale (MRC) scale is one of the most widely used tools for assessing muscle strength. It ranges from 0 to 5, with the possibility of using intermediate values (+/−) for greater sensitivity.

#### 2.1.2 Gait analysis

Gait analysis is a multidimensional instrumental examination that allows for quantitative and objective analysis of walking through the integrated collection of spatial-temporal, kinematic, dynamic, and electromyographic data. This organization provides a multifactorial, three-dimensional, non-invasive description of gait. The system used for the assessment is the SMART-DX4000, BTS Bioengineering (Milan), consisting of eight infrared cameras that allow the detection of joint kinematics data. A system of four force platforms enabled the detection of the ground reaction force, which, together with kinematic information, allowed the acquisition of data related to joint force moments. Finally, a surface electromyography system using wireless probes allowed the detection of the electrical activity of the muscles involved. The integration of the different modules ensures an objective and reproducible approach, useful for monitoring the typical alterations of neuromuscular pathologies and for evaluating the impact of surgical and rehabilitative treatments.

### 2.2 Focal muscle vibration treatment

Focal muscle vibration (fMV) was delivered using a dedicated device (Cro®System, Nemoco srl, Italy), consisting of an electromechanical transducer, a mechanical support and an electronic control unit. The mechanical support allowed the transducer to be oriented, positioned and fixed stably in relation to the patient's body, ensuring precise and repeatable application of the stimulus. The transducer was placed perpendicular to the belly of the target muscle. The pressure exerted by the mechanical support compressed the soft tissues overlying the muscle-tendon complex, producing a sinusoidal peak displacement of 0.1 mm at a constant frequency of 100 Hz. The reduced amplitude and high frequency were selected to specifically stimulate the primary afferents of the neuromuscular spindles (Ia fibres), while avoiding unwanted activation of the tonic vibration reflex.

Each session consisted of a series of 10-min stimulations interspersed with one minute of rest. The vibration, with low amplitude (0.1 mm) and a frequency of 100 Hz, was applied to the bellies of the quadriceps femoris, iliopsoas, rectus abdominis, quadratus lumborum, knee flexors, gluteus maximus and triceps surae muscles bilaterally.

Based on a new protocol following the Wolpaw research (11), patient was firstly treated in March 2025 for 6 consecutive days; in July 2025, the patient returned for a second cycle. In that occasion, in order to prolong the therapeutic effect, we decided to extend the number of sessions to a total of 15 fMV sessions over 15 consecutive days. Each area was treated with a single session, with the exception of the quadriceps femoris muscles (treated with 2/3 sessions already in the first cycle) and, during the second cycle, also the gluteal muscles and triceps surae (treated with 2 sessions).

## 3 Results

In Table 1 the results obtained with scales are described. FAS scale at T0 showed a total score of 21 (11 for physical fatigue and 10 for mental fatigue) and at T4 (after the second cycle of vibration) a reduction to 17 points was observed, with an improvement in both the physical and mental dimensions of fatigue.

**Table 1:** Clinical scale score.

Scales	T0	T4
FAS total score	21	17
FAS Mental state	10	8
FAS Physical State	11	9
SF-36 PF	45	60
SF-36 RP	40	50
SF-36 RE	66	100
SF-36 VT	70	70
SF-36 MH	88	88
SF-36 SF	75	100
SF-36 BP	55	65
SF-36 GH	60	75

The quality-of-life evaluation using SF-36 was analyzed at T0 and T4. The data showed a reduction in physical functioning at baseline (45/100), with significant limitations in more stressful activities, such as lifting weights and climbing stairs. Role limitations due to physical problems were present associated to emotional problems, reduced social functioning and pain perception; globally general health was at 60/100. At T4, improvement was observed in several domains: physical functioning, emotional role limitations social functioning perceived as complete and overall health to 75%. These results suggest a subjective perception of improved quality of life, although energy and emotional well-being remained stable.

Evaluation of gait analysis (see Table 2) showed that the gait cycle of the patient was characterized by an increase of the stance phase, an increased double support phase associated to a reduced cadence and reduced cycle length % height and compared to normal physiological values. At time T1, immediately after the end of the first treatment cycle (6 sessions), the data collected showed a slight improvement in the stance phase, a double support phase almost normalized while cadence and speed remained unchanged; a slight improvement in cycle length as a percentage of height was shown.

**Table 2:** Gait analysis parameters evaluated.

Temporal parameters	T0		T1		T2		T3		T4		T5	
	Adx	Asn	Adx	Asn	Adx	Asn	Adx	Asn	Adx	Asn	Adx	Asn
Cycle duration (s)	1.26 ± 0.01	1.28 ± 0.00	0.28 ± 0.01	1.27 ± 0.02	1.36 ± 0.03	1.35 ± 0.04	1.33 ± 0.02	1.35 ± 0.04	1.3 ± 0.02	1.37 ± 0.07	1.33 ± 0.06	1.17 ± 0.31
Support duration (s)	0.84 ± 0.02	0.88 ± 0.00	0.80 ± 0.03	0.82 ± 0.02	0.79 ± 0.01	0.84 ± 0.00	0.85 ± 0.05	0.85 ± 0.05	0.79 ± 0.03	0.82 ± 0.09	0.83 ± 0.03	0.82 ± 0.06
Oscillation duration (s)	0.47 ± 0.02	0.45 ± 0.01	0.51 ± 0.00	0.48 ± 0.03	0.56 ± 0.04	0.52 ± 0.01	0.53 ± 0.04	0.5 ± 0.03	0.51 ± 0.01	0.54 ± 0.04	0.5 ± 0.03	0.51 ± 0.02
Support phase (%)	66.43 ± 1.97	68.68 ± 0.46	62.09 ± 1.79	64.58 ± 1.25	58.35 ± 2.03	62.49 ± 1.63	60.38 ± 3.11	63.18 ± 2.68	60.75 ± 1.59	60.1 ± 4.00	62.12 ± 1.0	76.06 ± 24.51
Oscillation phase (%)	37.14 ± 1.14	35.41 ± 0.25	39.59 ± 0.41	37.51 ± 1.89	41.65 ± 2.03	38.67 ± 0.26	39.62 ± 3.11	36.82 ± 2.68	39.25 ± 1.59	39.9 ± 4.00	37.88 ± 1.0	48.18 18.39
Single support phase (%)	35.97 ± 0.03	36.57 ± 1.41	37.22 ± 1.99	39.9 ± 0.48	38.38 ± 0.11	41.96 ± 1.88	37.35 ± 2.55	39.07 ± 3.33	41.76 ± 2.30	37.37 ± 1.07	38.3 ± 1.1	47.37 ± 17.09
Dual support phase (%)	16.62 ± 1.78	14.4 ± 0.44	12.51 ± 1.75	12.06 ± 0.45	11.7 ± 3.76	8.73 ± 0.33	12.54 ± 1.59	10.56 ± 3.85	10.80 ± 2.04	10.25 ± 2.57	12.55 ± 2.9	13.87 ± 4.61
Average speed (m/s)	0.9 ± 0.00		0.9 ± 0.00		0.9 ± 0.00		0.9 ± 0.00		0.9 ± 0.00		0.8 ± 0.01	
Average speed (% height/s)	49.3 ± 0.68		51.92 ± 1.59		49.27 ± 0.23		48.97 ± 1.87		51.07 ± 0.75		46.32 ± 5.70	
Cadence (steps/minute)	94.2 ± 0.60		94 ± 1.233		89.1 ± 2.10		89.6 ± 1.855		90.2 ± 2.315		101.7 ± 21.619	

Spazial parameters	T0		T1		T2		T3		T4		T5	
	Adx	Asn	Adx	Asn	Adx	Asn	Adx	Asn	Adx	Asn	Adx	Asn
Cycle length (m)	1.14 ± 0.02	1.13 ± 0.03	1.21 ± 0.02	1.17 ± 0.03	1.20 ± 0.03	1.21 ± 0.01	0.18 ± 0.03	1.18 ± 0.02	1.2 ± 0.01	1.24 ± 0.01	1.20 ± 0.02	1.05 ± 0.25
Cycle length (% height)	63.51 ± 1.13	62.99 ± 1.39	67.32 ± 1.16	65.18 ± 1.51	66.48 ± 1.47	67.01 ± 0.56	66.59 ± 1.52	65.79 ± 1.1	66.86 ± 0.63	69.08 ± 0.82	66.54 ± 0.95	58.15 ± 13.71
Step length (m)	0.56 ± 0.02	0.58 ± 0.00	0.59 ± 0.01	0.61 ± 0.02	0.58 ± 0.04	0.61 ± 0.02	0.59 ± 0.02	0.59 ± 0.01	0.62 ± 0.01	0.60 ± 0.01	0.59 ± 0.01	0.61 ± 0.01
Step width (m)	0.22 ± 0.01		0.22 ± 0.01		0.20 ± 0.00		0.21 ± 0.01		0.20 ± 0.02		0.21 ± 0.01	

Two months after the end of the first treatment cycle, at time T2, two key elements emerged: the cycle duration increased compared to T1, indicating that the progress previously achieved had been lost, and the asymmetry between the right and left sides increased, with better control observed of the right side.

At time T3, approximately 4 months after the end of the first treatment cycle and immediately before the start of the second cycle, the step cycle duration was increased compared to T1, indicating that the progress made had been largely lost. Step length, which had improved from T0 to T1, worsened.

At time T4, the improvements achieved at T1 are reproduced with a further small increase in benefits and good maintenance of progress after two months (T5).

At time T5, the data show: An increased cadence compared to all other tests performed with a normal step length. All improvements obtained at T1 are confirmed at T4 and remain until T5.

Overall, between T0 and T5, a progressive normalization of the global walking parameters was observed: speed increases, cycle length increases, and the stance and double support phases were reduced. The general pattern became more symmetrical and functional, indicating a favorable evolution of motor control.

**Figure 1:**
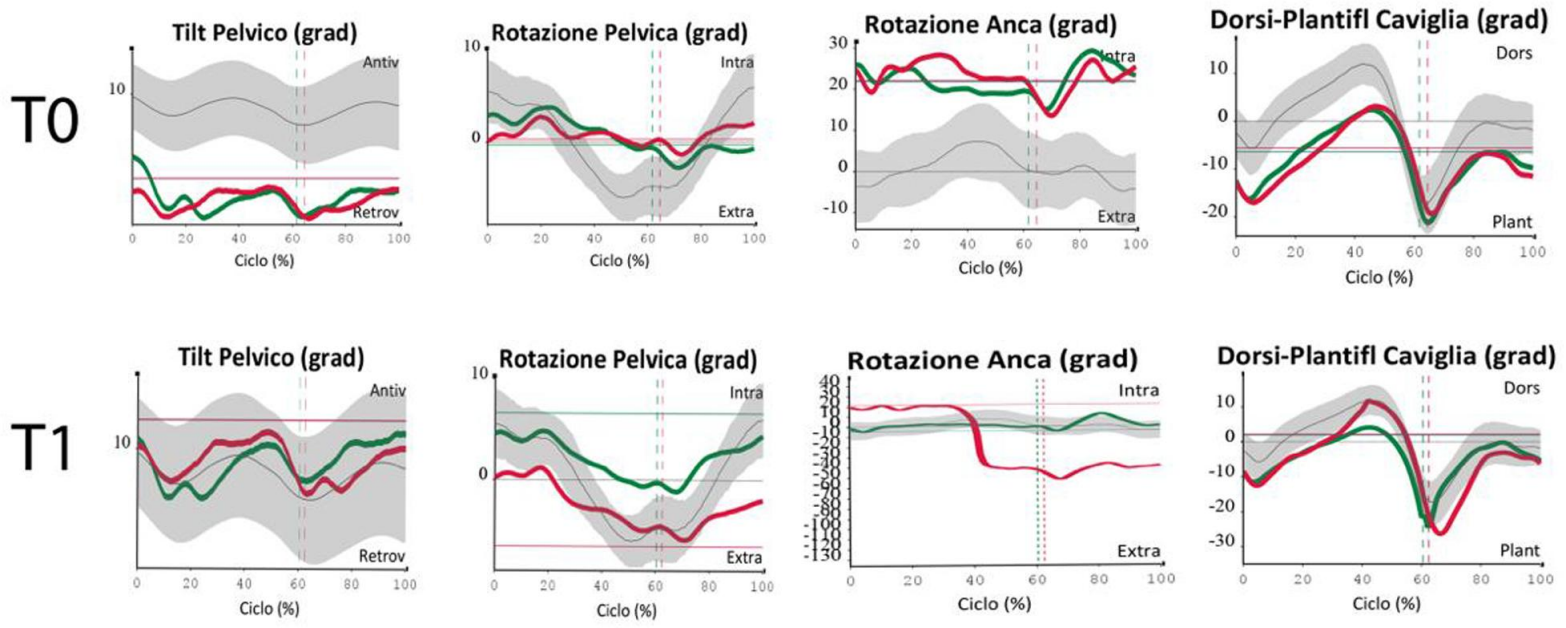
Hip internal rotation, pelvic retroversion, reduced range of motion in ankle dorsiflexion at T0 and T1.

The joint assessment of kinematics and dynamics between T0 and T5 documents a harmonious recovery of gait mechanics, with a reduction in pelvic compensation, improved joint sequence and a more efficient and physiological distribution of forces. Specifically, at time T0, the gait pattern shows the following kinematic and dynamic alterations: retroverted pelvis, internally rotated hip, reduced joint range of motion in ankle dorsiflexion (plantarflexion gait), reduced knee flexion in the load acceptance phase, reduction in knee extension and hip flexion moments. The pattern resulting at T1, after the first treatment cycle, is characterized by stability, symmetry and fluidity of the parameters: the pelvis is no longer retroverted, there is improvement in pelvic rotation, improvement in ankle dorsiflexion and knee extension and hip flexion moments. These benefits were maintained until follow-up (T5), resulting in more economical and safer walking (see Figure 1).

Surface electromyographic analysis conducted at different assessment times (T0–T5) allowed the evolution of muscle activation patterns in the gait cycle to be monitored, highlighting significant changes in neuromotor control and coordination following proprioceptive treatment.

At time T0, an altered activation pattern was detected. The right tibialis anterior showed persistent activity even during the stance phase, when it should physiologically be inactive, indicating a loss of motor selectivity and a possible compensatory mechanism. The left rectus femoris was inactive, while the right rectus femoris showed co-contraction activity even during the stance phase.

At time T1, a marked improvement in muscle recruitment and coordination was observed. The right tibialis anterior showed more physiological activation, with early shutdown compared to the stance phase. Both the right and left rectus femoris showed a more regular activation pattern, with reduced co-contraction activity. The right semitendinosus also showed more synchronized activity.

At time T2, two months after treatment (in line with the 40 days of the Wolpaw protocol (12), and even more so at T3 (four months after treatment), there is a worsening in the activation pattern, especially in the rectus femoris muscles.

After the second cycle of 15 mechanical vibration sessions (time T4), a significant recovery and a good response to proprioceptive treatment was observed compared to T3. The left rectus femoris, in particular, returned to correct functioning in the initial phase of the gait cycle, re-establishing a more physiological pattern. The semitendinosus muscles showed a reduction in co-activation. Overall, the electromyographic pattern appears more harmonious and functional.

Finally, at time T5, the benefits acquired by the tibialis anterior were confirmed, maintaining regular activation consistent with the physiology of the step. The rectus femoris muscles showed a slight reduction in activity compared to T4, suggesting improved motor control which was acquired thanks to the increased number of sessions compared to the first cycle. The semitendinosus, on the other hand, showed new coactivation, indicating a partial return to compensatory strategies.

**Figure 2:**
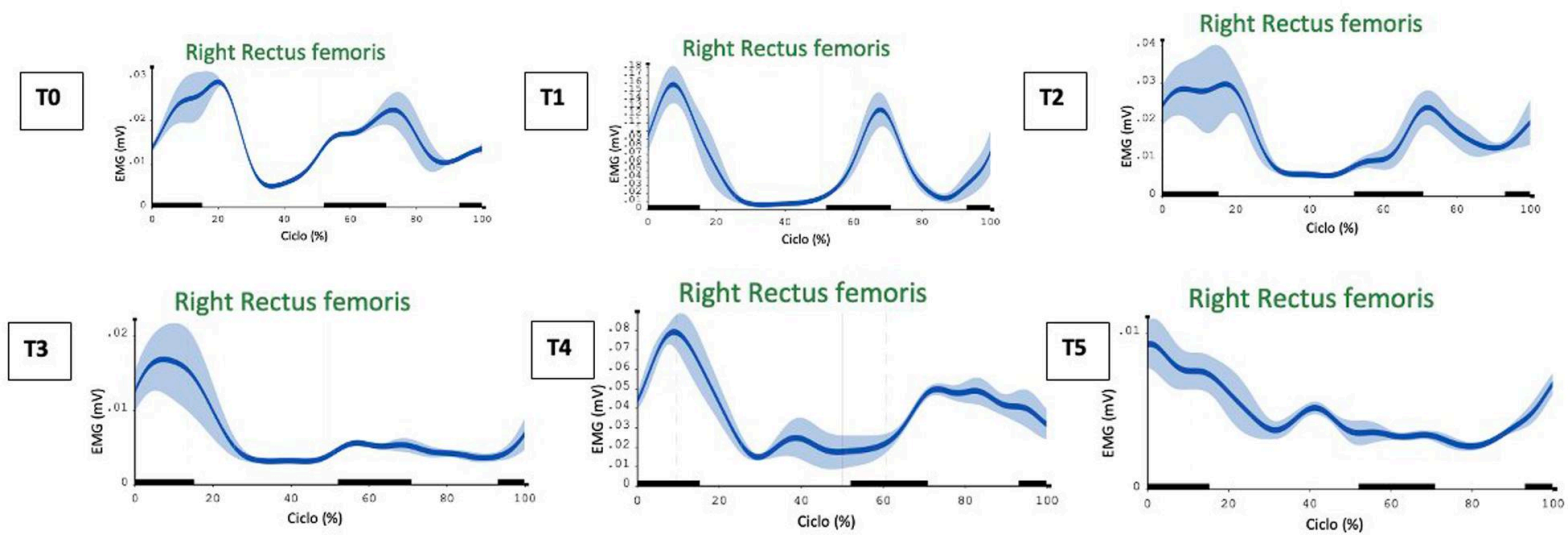
Muscle activity of the rectus femoris during the different evaluations.

Focusing on the most compromised muscle (rectus femoris), we analyzed the electromyographic data: at T0, we note abnormal control. The activation pattern improved at T1 after the first cycle of 6 sessions and gradually worsened until T3. However, at T5, we can see good maintenance of progression 2 months after the second cycle of 15 sessions (see Figure 2).

## 4 Discussion

The clinical experience described in this paper highlights how the integration of innovative approaches such as the use of focal muscle vibration within the rehabilitation program for patients with Becker muscular dystrophy (BMD) can be an effective strategy for improving motor function and quality of life. This study is limited to a single case and, although results obtained are encouraging they should be seen as a hypothesis-generating observation that need to be confirmed by larger population study.

Becker muscular dystrophy presents significant clinical variability, which complicates therapeutic and rehabilitation management. In this context, the possibility of acting on neuromotor control becomes a central factor. In the clinical case analyzed, treatment with mechanical vibration led, through proprioceptive stimulation (13, 14), to a perceived improvement in the strength and endurance of the lower limbs, associated with a significant reduction in instability and the frequency of falls. In terms of ecological rehabilitation, we observed a significant improvement in climbing and descending stairs thanks to the recovery of a motor pattern free of the obvious compensations that were present before treatment, especially when climbing. After the first cycle of six sessions, the patient maintained the benefit for about two months, with a stability of improvement at time T2. The second cycle, consisting of fifteen sessions, improved the quality of movement and prolonged its duration over time, with an improvement after two months from the end of treatment. Surface electromyographic analysis and gait parameters confirmed a functional recovery process characterized by phases of improvement and regression, suggesting the need for periodic and prolonged proprioceptive rehabilitation protocols associated with concomitant physiotherapy.

Overall, the data collected require further study in a large population using this protocol to better understand the role of focal muscle vibration as a possible rehabilitation tool in the management of Becker's dystrophy, complementing traditional physiotherapy strategies and emerging pharmacological and genetic approaches, including the possible effect related to acquisition of skills from repetitive learning.

## 5 Conclusions

Focal muscle vibration could constitute a new rehabilitation approach, based also on translational medicine studies in the management of patients with Becker muscular dystrophy, considering that to the best of our knowledge, at this moment there aren't specific therapies identified to be useful for this pathology. Studies on a large cohort of subjects with the same characteristics should be necessary to confirm these results.

## Data Availability

The raw data supporting the conclusions of this article will be made available by the authors, without undue reservation.
